# Supplementary material for: Electroanalysis Applied to Compatibility and Stability Assays of Drugs: Carvedilol Study Case
Source: Pharmaceuticals (Basel). 2020 Apr 17;13(4):70. doi: 10.3390/ph13040070 (PMC7243106; doi:10.3390/ph13040070)
Supplement: Supplementary file 1 [file pharmaceuticals-13-00070-s001.pdf]

Table 1. Statistical analysis of the degration profile of the tested oils.

Room temperature degration (25°)					
	**Mean**	**SD**	**Variance**	**Chi-Square**	**Prob. H0***
**CP control**	90.3092	16.7777	281.4917	-	-
**Plurol**	91.2157	14.7687	218.1152	3.09944	0.91735
**Estearic Acid**	92.5201	11.7570	138.2288	1.96424	0.51533
**Canola**	78.6005	22.4953	506.0421	7.19091	0.25227
**Safflower Oil**	78.8916	14.8963	221.9022	3.15325	0.93497
**Forced degration (50°)**					
	**Mean**	**SD**	**Variance**	**Chi-Square**	**Prob. H0***
**CP control**	91.1374	15.3919	236.911	-	-
**Plurol**	109.4842	30.3605	921.7638	15.5631	0.00733
**Estearic acid**	75.7983	15.7040	246.6163	4.16388	0.76856
**Oleic acid**	91.0320	6.4916	42.14192	0.71153	0.1002
**Canola oil**	77.0923	16.4737	271.3842	4.58206	0.66585

*Null Hypothesis (H0): the variances are equal to CP control variance
